# A Practical Treatise on Materia Medica and Therapeutics, with Especial Reference to the Clinical Application of Drugs

**Published:** 1907-01

**Authors:** 


A PRACTICAL TREATISE ON MATERIA MEDICA AND THERAPEUTICS, WITH ESPECIAL REFERENCE TO THE CLINICAL APPLICATION OF DRUGS. By John V. Shoemaker, M. D., LL. D., Professor of Materia Medica, Pharmacology, Therapeutics, and Clinical Professor of Diseases of the Skin in the Medico-Chirurgical College of Philadelphia; Physician to the Medico-Chirurgical Hospital; Member of the American Medical Association and the British Medical Association; Fellow of the Medical Society of London, etc., etc. Sixth edition. Thoroughly revised. (In conformity with latest revised U. S. Pharmacopeia, 1905.) Royal Octavo, 1244 pages. Extra cloth, price, $5, net; full sheep, price, $6, net. F. A. Davis Company, Publishers, 1914-16 Cherry St., Philadelphia, Pa.

Shoemaker's Materia Medica and Therapeutics is very popular with physicians and students, and the author is equally and deservedly popular. The book is too well known, and has been a familiar text-book too long to require more than a mention that the sixth edition, carefully revised and in excellent shape, is now ready for delivery. It is standard and will be the text-book for many generations. , DR. DANIEL.

